# Spatio-Temporal Joint Network for Coupler Anomaly Detection Under Complex Working Conditions Utilizing Multi-Source Sensors

**DOI:** 10.3390/s26092661

**Published:** 2026-04-24

**Authors:** Zhirong Zhao, Zhentian Jiang, Qian Xiao, Long Zhang, Jinbo Wang

**Affiliations:** 1 Rolling Stock Branch, CHN Energy Shuohuang Railway Development Co., Ltd., Cangzhou 062350, China; zhirong.zhao.a@ceic.com (Z.Z.); 11074809@ceic.com (Z.J.); 2 School of Mechatronics and Vehicle Engineering, East China Jiaotong University, Nanchang 330013, China; jxralph@ecjtu.edu.cn (Q.X.); wwwbojw@163.com (J.W.)

**Keywords:** heavy-haul train, coupler and draft gear system, normalized mutual information, spatio-temporal graph neural network, anomaly detection

## Abstract

Owing to the intricate mechanical coupling characteristics and the considerable difficulty in extracting synergistic spatio-temporal features from high-dimensional sensor data under fluctuating alternating loads, this study proposes a robust anomaly detection framework that combines Normalized Mutual Information (NMI) and Spatio-Temporal Graph Neural Networks (STGNN). First, NMI is utilized to quantify the nonlinear physical coupling intensity among multi-source sensors, thereby filtering out weakly correlated noise and reconstructing the spatial topological structure of the coupler system. Subsequently, a deep learning architecture incorporating Graph Convolutional Networks (GCN), Gated Recurrent Units (GRU), and one-dimensional convolutional residual connections is developed to capture the dynamic evolutionary characteristics of equipment states across both spatial interactions and temporal sequences. Finally, based on the model’s health-state predictions, a moving average algorithm is introduced to smooth the residual sequences, and an anomaly early-warning baseline is established in conjunction with the 3σ criterion. Experimental validation conducted using field service data from heavy-haul trains demonstrates that, compared to conventional serial CNN and Long Short-Term Memory (LSTM) models, the proposed method exhibits superior fitting performance and robustness against noise, effectively reducing the false alarm rate within normal working intervals. In a real-world case study, the method successfully identified variations in spatial linkage features induced by local damage and triggered timely alerts. Notably, the spatial alarm nodes were highly consistent with the fatigue crack initiation sites identified through on-site magnetic particle inspection. This study provides a viable data-driven analytical framework for the condition monitoring and anomaly identification of critical load-bearing components in heavy-haul trains.

## 1. Introduction

Concomitant with the evolution of heavy-haul railways toward increased axle loads and extended train configurations, the service safety and structural integrity of critical load-bearing components, such as coupler and draft gear systems, under complex alternating loads have garnered significant attention [1]. Traditional preventive maintenance protocols and manual offline non-destructive testing (NDT) are constrained by operational inefficiency and an inability to capture latent risks in a real-time manner [2]. Consequently, transitioning toward condition-based maintenance (CBM) supported by real-time sensor data has emerged as an imperative strategy for enhancing maintenance precision and ensuring operational reliability [3]. Nevertheless, the mechanical stress and deformation mechanisms inherent in coupler systems are highly sophisticated, but existing data-driven fault diagnosis methodologies predominantly focus on univariate sensor time-series analysis. When applied to dense sensor networks within coupler systems, these approaches struggle to fully characterize the potent nonlinear spatial coupling and dynamic latency features among multi-dimensional signals. This limitation frequently leads to missed detections of incipient faults under complex working conditions, while also increasing susceptibility to false alarms triggered by high-frequency noise interference [4].

In recent years, extensive scholarly efforts have been dedicated to the condition monitoring and assessment of critical mechanical components during service. Early investigations primarily relied on physical failure mechanisms and finite element method (FEM) simulations [5]. Zhang et al. [6] conducted systematic experimental research on the dynamic performance and compressive stability of heavy-haul locomotives under longitudinal forces through a series of field tests involving 10,000-ton trains. By establishing a numerical model that accounts for complex nonlinear contact, Ren et al. [7] predicted that the remaining useful life (RUL) of a 4 mm initial surface crack within a coupler is approximately 700,000 km. Furthermore, Dumitriu [8] analyzed the vertical vibrational responses of bogies by integrating experimental data with rigid–flexible coupling numerical simulations, thereby exploring the feasibility of fault detection methodologies for primary suspension dampers.

While physical-mechanism-based studies offer high fidelity under specific, well-defined conditions, the actual working environments and loading regimes of heavy-haul trains are exceedingly complex. Purely physical models struggle to accurately characterize nonlinear degradation signatures under multi-source variable-amplitude loading. Given these intricate evolutionary patterns, such models often exhibit insufficient precision when describing service states, ultimately failing to satisfy the rigorous demands of practical engineering applications [9].

To address these challenges, researchers have increasingly pivoted toward data-driven methodologies leveraging real-time monitoring data, which has progressively emerged as a focal point of scholarly inquiry in this field [10]. Early data-driven investigations were primarily predicated upon conventional shallow machine learning algorithms. Li et al. [11] integrated wavelet packet decomposition (WPD) with one-class support vector machines (OCSVM) to design an Internet of Things (IoT)-based condition monitoring system for mechanical equipment, facilitating the effective identification of anomalies through signal feature extraction. Similarly, the study conducted by Li et al. [12] incorporated classical machine learning algorithms, such as k-nearest neighbors (k-NN) and clustering, to refine the Multivariate State Estimation Technique (MSET), achieving efficient anomaly pattern recognition by constructing health data intervals. Gómez et al. [13] leveraged WPD to extract energy features from axle-box vibration signals and, coupled with SVM, realized high-reliability real-time diagnostics for railway axle cracks. Furthermore, Dhiman et al. [14] utilized Neighborhood Component Analysis (NCA) for feature selection, combining support vector regression (SVR) with decision tree models to achieve high-precision state prediction for critical wind turbine components.

Nevertheless, traditional machine learning approaches exhibit a profound reliance on domain expertise and manual feature engineering [15]. When confronted with the massive, high-dimensional, and nonlinear monitoring datasets generated by heavy-haul trains under complex working conditions, shallow-architecture networks often encounter significant impediments, such as suboptimal feature extraction and inadequate generalization capabilities [16]. These inherent architectural constraints hinder the effective discernment of latent underlying patterns within complex scenarios, resulting in insufficient adaptability within authentic field service environments [17].

To circumvent reliance on manual prior knowledge, deep learning architectures characterized by self-adaptive capabilities have established dominance in the field of condition monitoring. By facilitating automated feature extraction, this paradigm demonstrates superior adaptability when processing massive datasets [18]. Addressing the challenge of limited fault sample acquisition, Liu et al. [19] developed an enhanced autoencoder model integrating CNN and LSTM. By leveraging the frequency-domain characteristics of normal samples, they achieved accurate early warning for unknown faults and significantly bolstered the model’s generalization capability in complex industrial environments. Li et al. [20] proposed an unsupervised anomaly detection methodology fusing stacked autoencoders and LSTM, which incorporates WPD to extract multi-feature sequences, enabling effective condition recognition for rotating machinery using entirely unlabeled historical data. Furthermore, Hu et al. [21] introduced a reconstruction approach termed ADMM-1DNet, based on the synthesis of deep neural networks (DNN) and compressed sensor. By mapping the Alternating Direction Method of Multipliers (ADMM) onto a deep network architecture and learning redundant analysis operators, they effectively enhanced the reconstruction precision and feature-preservation capacity of vibration signals under high-noise backgrounds. Exploring non-contact acoustic monitoring scenarios, Zou et al. [22] proposed a multi-channel few-shot learning framework that utilizes a global optimization layer strategy to overcome the challenges of anomaly detection with limited samples in complex environments. Additionally, Hu et al. [23] developed a forecasting framework combining time-series autocorrelation decomposition with CNN, which significantly refines RUL prediction accuracy by deeply integrating both long- and short-term health state features.

Existing deep learning architectures predominantly treat multi-dimensional monitoring signals as independent sequences, prioritizing the extraction of temporal features while neglecting the spatial physical topology dictated by mechanical structures and force transmission pathways [24]. Under the complex loading regimes of heavy-haul trains, this disregard for spatial collaborative constraints compromises the sensitivity to incipient anomalies and increases susceptibility to false alarms triggered by local noise interference [25].

To circumvent the bottleneck of synergistically extracting spatio-temporal features from multi-dimensional sensor signals, Graph Neural Networks (GNN) have recently been introduced into the domain of complex equipment condition monitoring, owing to their unique advantages in processing non-Euclidean data and capturing intricate topological dependencies among nodes [26]. Liang et al. [27] proposed an end-to-end framework integrating Graph Attention Networks (GAT) and adaptive Transformers, effectively addressing the deficiencies of conventional models in overlooking multi-sensor spatial correlations. In response to the volatile working conditions of real-world scenarios, Fu et al. [28] developed a multi-scale dynamic STGNN that synergizes GAT mechanisms with long- and short-term temporal branches, significantly bolstering the dynamic adaptability and robustness of equipment anomaly detection. For high-dimensional and noisy industrial control data, Wang et al. [29] leveraged prior knowledge to construct dynamic graph models coupled with denoising modules, presenting a spatio-temporal graph anomaly detection framework suitable for fine-grained condition monitoring. To further enhance the credibility and physical consistency of deep learning models, Liu et al. [30] embedded domain-specific physical mechanism knowledge into GNNs, constructing physics-guided spatio-temporal models to achieve high-precision predictions. Furthermore, Wang et al. [31] innovatively extended graph modeling strategies to the frequency domain by constructing graph-mapped spectra reflecting the spatio-temporal dynamics of short-time periodogram spatial configurations, thereby providing a novel perspective for the degradation monitoring of bearing health states. Du et al. [32] developed a mechanism-embedded hybrid graph neural network to improve the generalization ability of anomaly detection in industrial cyber–physical systems under distribution shift and unknown operating conditions. Pan et al. [33] proposed a multi-node network-based blind deconvolution approach with adaptive frequency band segmentation to achieve effective fault feature extraction and fault detection for high-speed train axlebox bearings under low signal-to-noise ratio environments.

Addressing the challenges of topological graph construction and feature extraction, this study focuses on heavy-haul train coupler and draft gear systems and proposes an anomaly monitoring methodology termed NMI-STGNN. This scheme leverages NMI to quantify the correlations among sensors, thereby filtering noise interference while reconstructing the spatial topology. By integrating GCN and GRU, the model is capable of deeply mining the spatio-temporal evolutionary laws of equipment states. Based on prediction residuals and the 3σ criterion, the system adaptively establishes a health baseline, ensuring high sensitivity in anomaly monitoring. This research provides a reliable theoretical and practical reference for the intelligent operation and maintenance of critical components.

The primary contributions of this work are summarized as follows:

(1) A methodology leveraging NMI is proposed to quantify the nonlinear physical coupling intensity among multi-source sensors within the coupler system. This approach effectively eliminates weakly correlated redundant noise and reconstructs a spatial topological graph structure that faithfully reflects the force transmission and linkage characteristics between components, thereby providing reliable physical prior constraints for subsequent spatial feature extraction.
(2) An STGNN model integrating GCN and GRU is developed, incorporating one-dimensional convolutional residual connections. By overcoming the limitations of conventional univariate temporal models, this architecture enables the deep joint mining of spatial interactions and long-term temporal dynamic evolutionary characteristics of monitoring signals under complex alternating loads, significantly enhancing feature extraction capacity and prediction accuracy in high-frequency noise environments.
(3) An adaptive anomaly early-warning mechanism is designed based on moving average smoothing of prediction residuals and the 3σ criterion. Validated by authentic field service data from heavy-haul trains, this mechanism not only effectively reduces the false alarm rate under normal complex working conditions but also acutely and precisely captures variations in spatial linkage features induced by local fatigue cracks, such as knuckle damage, achieving high-reliability early anomaly diagnosis for critical components.

The remainder of this paper is organized as follows: Section 2 introduces the fundamental theories underlying GCN and GRU. Section 3 elaborates on the proposed NMI-STGNN anomaly monitoring methodology, including the construction of spatial correlation graphs, the design of the spatio-temporal joint feature extraction model, and the workflow of the adaptive early-warning mechanism. In Section 4, the prediction performance and anomaly warning capabilities of the proposed method are comprehensively validated and comparatively analyzed using authentic field operation data. Finally, Section 5 concludes the paper.

## 2. Preliminaries

### 2.1. Graph Convolutional Networks

**Figure 1:**
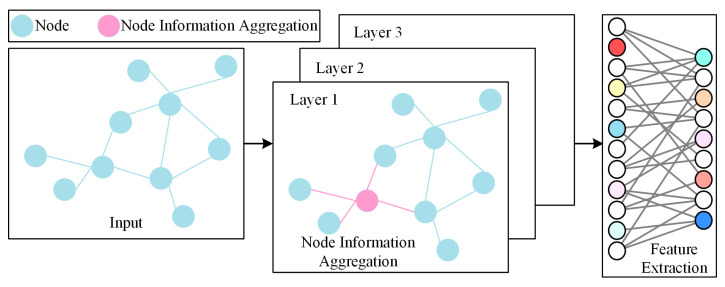
Graph convolutional network.

GCNs represent a sophisticated class of deep learning architectures engineered to operate directly on data structured as graphs [34]. Distinct from conventional CNNs optimized for Euclidean domains, GCNs are specifically designed to capture intricate topological relationships between nodes, as illustrated in Figure 1. The fundamental principle involves a simplified spectral decomposition of the graph Laplacian operator, which facilitates the aggregation and transformation of feature information from a node and its adjacent neighbors. Through the hierarchical stacking of multiple layers, the model can effectively extract high-order spatial representations of the constituent nodes within the network [35].

Prior to the execution of graph convolutions, an adjacency matrix incorporating self-connections, denoted as $\tilde{A}$, is defined to ensure that a node’s intrinsic information is preserved during the neighborhood feature aggregation process:

(1) $$\tilde{A}=A+I$$

where A represents the original adjacency matrix and I is the identity matrix.

Subsequently, a symmetrically normalized graph Laplacian matrix is constructed to facilitate information smoothing and spatial scaling within the spectral domain:

(2) $$\hat{L}={\tilde{D}}^{-\frac{1}{2}}\tilde{A}{\tilde{D}}^{-\frac{1}{2}}$$

where $\tilde{D}$ is the degree matrix of $\tilde{A}$.

Building upon these operators, the forward propagation rule for a GCN layer is defined as follows:

(3) $$H^{\left(l+1\right)}=f_{\rho }\left(\hat{L}H^{\left(l\right)}\Gamma^{\left(l\right)}\right)$$

where $H^{\left(l\right)}$ denotes the node feature matrix of the *l-th* layer, $\Gamma^{\left(l\right)}$ represents the matrix of trainable weights, and $f_{\rho }\left(\cdot \right)$ signifies the non-linear activation function.

### 2.2. Gated Recurrent Units

**Figure 2:**
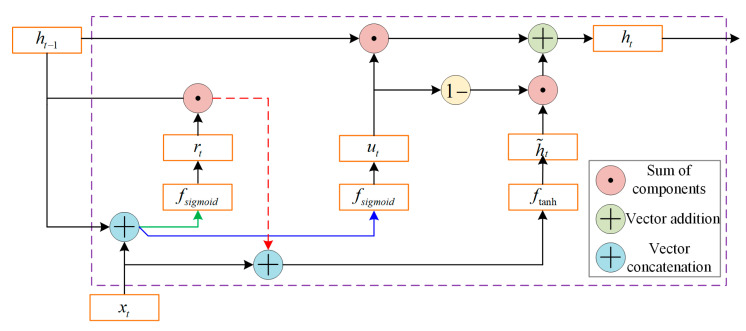
Schematic diagram of the internal GRU structure.

The GRU is a significant variant of RNNs, specifically engineered for processing time-series data characterized by long-term dependencies [36]. To alleviate the gradient vanishing problem inherent in standard RNNs, the GRU incorporates a gating mechanism to meticulously regulate the flow of information, as illustrated in Figure 2. Compared to LSTM, the GRU architecture is more streamlined. By consolidating the forget and input gates into a single update gate, it effectively reduces the parameter count while preserving the robust capacity for complex temporal modeling.

At each time step t, the GRU initially computes two gating signals—the update gate $u_{t}$ and the reset gate $r_{t}$—based on the current input $x_{t}$ and the hidden state from the previous time step $h_{t-1}$:

(4) $$\left\{\begin{array}{l} u_{t}=f_{sigmoid}\left(\omega_{u}\cdot \left[h_{t-1},x_{t}\right]+b_{u}\right)\\ r_{t}=f_{sigmoid}\left(\omega_{r}\cdot \left[h_{t-1},x_{t}\right]+b_{r}\right) \end{array}\right.$$

Specifically, the update gate $u_{t}$ determines the proportion of information from the previous state to be retained in the current state, while the reset gate $r_{t}$ regulates the extent to which the previous state influences the current candidate information.

The state update logic then utilizes the reset gate signal to compute the candidate hidden state ${\tilde{h}}_{t}$ for the current step:

(5) $${\tilde{h}}_{t}=f_{\tanh}\left(\omega_{h}\cdot \left[r_{t}⊙h_{t-1},x_{t}\right]+b_{h}\right)$$

Finally, a linear interpolation between the historical state $h_{t-1}$ and the candidate state ${\tilde{h}}_{t}$ is performed via the update gate $u_{t}$ to derive the output state $h_{t}$ for the current time step:

(6) $$h_{t}=\left(1-u_{t}\right)⊙h_{t-1}+u_{t}⊙{\tilde{h}}_{t}$$

## 3. Proposed Method

To effectively characterize the intricate spatio-temporal coupling patterns among the sensors within the coupler system, this study proposes an anomaly detection methodology integrating NMI and STGNN. The proposed approach encompasses three pivotal phases: spatial graph construction, joint spatio-temporal feature extraction, and smoothed residual-based early warning.

### 3.1. Spatial Graph Construction via NMI

**Figure 3:**
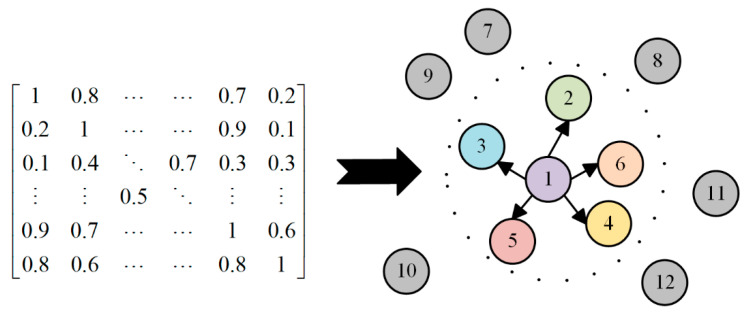
Construction of the coupler sensor association matrix and spatial topological graph based on NMI.

To address the nonlinear coupling characteristics of coupler sensor data under complex working conditions, this study employs NMI instead of the linear correlation coefficient for association measurement. By quantifying high-order correlations between nodes, the topological adjacency matrix of the GNN is reconstructed to achieve precise modeling of the spatial structural features of the coupler system, as illustrated in Figure 3.

Let X and Y represent the time-series monitoring data from two distinct sensors. Their mutual information I(X,Y) is defined as:

(7) $$I(X,Y)=\iint p(x,y)\log\frac{p(x,y)}{p(x)p(y)}dxdy$$

where p(x,y) denotes the joint probability density function of X and Y, while p(x) and p(y) are their respective marginal probability density functions. To eliminate the influence of varying information entropy magnitudes across different sensors on the association measurement, a symmetric normalization is applied to the mutual information:

(8) $$NMI(X,Y)=\frac{I(X,Y)}{\sqrt{H(X)H(Y)}}$$

where H(X) and H(Y) represent the information entropy of variables X and Y, respectively. The off-diagonal elements of the calculated NMI matrix are strictly distributed within the interval [0, 1], where higher values signify more potent physical coupling between the sensors.

To avert interference from weakly correlated noise during node aggregation, a Top-k sparsification strategy is applied to the fully connected NMI matrix. For each sensor node, only the *K* neighbors with the highest association degrees are retained in this study, *K* = 5, yielding the final sparse binary adjacency matrix A:

(9) $$A_{ij}=\left\{\begin{array}{l} 1,NMI\left(X_{i},X_{j}\right)\in Top-k\left(X_{i}\right)\\ 0,otherwise \end{array}\right.$$

This adjacency matrix A explicitly characterizes the linkage topological relationships within the internal mechanical structure of the coupler, providing structural support for subsequent spatial feature extraction.

This study adopts NMI to construct the graph structure, whose core logic is to mine the dynamic correlations hidden behind sensor signals through statistical methods, endowing the data-driven topological structure with clear physical significance. During the service of the heavy-haul train coupler and draft gear system, longitudinal impact is transmitted through core components such as coupler tongues, coupler bodies, and draft gears. This mechanical interaction, affected by mechanical clearance, frictional contact, and nonlinear damping of the draft gear, exhibits significant nonlinear characteristics. Compared with traditional linear correlation coefficients, NMI can more accurately capture such complex high-order coupling relationships, where high mutual information values directly correspond to the main stress propagation paths in the system or mechanical components with close physical connections. The sparse adjacency matrix generated by retaining strong correlation connections essentially constructs a virtual framework reflecting the mechanical topology of the coupler system for the model. In the subsequent spatial feature aggregation process, graph convolution operations can simulate the diffusion process of mechanical energy in the physical structure, ensuring that the high-dimensional features extracted by the model are always constrained by the underlying mechanical connection logic. This design not only improves the model’s sensitivity to early faint damage but also establishes a strong correlation between the originally black-box deep learning process and the actual force-bearing behavior of the system.

### 3.2. Spatio-Temporal Graph Neural Network Model

**Figure 4:**
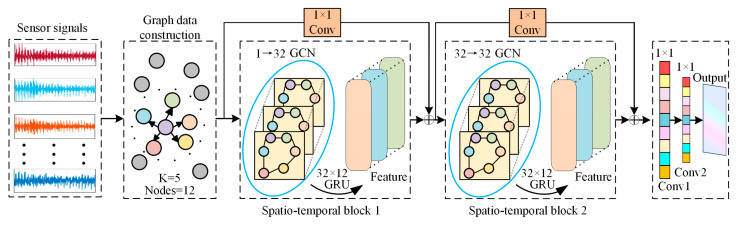
Overall architecture of the NMI-STGNN model.

Building upon the reconstructed spatial topology, this study engineered an STGNN model comprising GCN, GRU, and 1D-CNN residual connections to facilitate the joint mining of spatio-temporal features from equipment states, as illustrated in Figure 4. The specific architectural parameters of the model are summarized in Table 1. At any time step t within the sliding window, the GCN leverages the adjacency matrix A to aggregate features from sensor nodes and their respective neighbors, enabling the interaction of spatial physical information. The forward propagation process of a single GCN layer is formulated as:

(10) $$H^{\left(l+1\right)}=f_{relu}\left(AH^{\left(l\right)}W^{\left(l\right)}\right)$$

where $H^{\left(l\right)}$ denotes the feature representation of nodes in the *l-th* layer, $W^{\left(l\right)}$ is the trainable weight matrix for the current layer, and $f_{relu}\left(\cdot \right)$ represents the ReLU activation function. Through matrix multiplication, the model implicitly captures the mechanical response transmission characteristics among adjacent sensors.

To characterize the dynamic degradation patterns of equipment states over time, the spatial feature sequences extracted by the GCN are fed into the GRU network. The GRU models long-term temporal dependencies within the sliding window—set to a length of 60 in this study—via its update and reset gate mechanisms. Simultaneously, to mitigate feature degradation and gradient vanishing inherent in deep architectures, 1D-CNN residual skip connections are integrated into the spatio-temporal blocks. The fusion output mechanism of the entire spatio-temporal block is described as:

(11) $$f_{out}=f_{relu}(f_{GRU}(f_{GCN}(X,A))+f_{conv1d}(X))$$

This configuration preserves fine-grained temporal fluctuations from the raw sensor data while extracting high-order spatio-temporal coupling features.

The primary model consists of two stacked spatio-temporal blocks. The architecture concatenates the features from the final time step of the original input with the high-level spatio-temporal features, followed by two 1D-CNN layers for dimensionality reduction mapping to output the predicted values $\hat{Y}$ for each sensor at the next time step. The model is trained using MSE as the loss function:

(12) $$L_{MSE}=\frac{1}{B\cdot N}\sum_{B}^{b=1}\sum_{N}^{i=1}{\left(Y_{b,i}-{\hat{Y}}_{b,i}\right)}^{2}$$

where B denotes the batch size and N represents the number of sensor nodes.

### 3.3. Adaptive Early-Warning Mechanism Based on Smoothed Residuals and the 3σ Criterion

During the training phase, the model exclusively captures the data distribution characteristic of normal working conditions. In the monitoring stage, when structural anomalies such as fatigue cracks or component loosening occur within the coupler system, the resultant physical responses diverge from the established normal linkage patterns, leading to a pronounced escalation in prediction residuals.

The composite residual intensity at time t is quantified as the Root Mean Square Error (RMSE) of the prediction deviations across all core sensors:

(13) $$E_{t}=\sqrt{\frac{1}{N}\sum_{N}^{i=1}{\left(Y_{i,t}-{\hat{Y}}_{i,t}\right)}^{2}}$$

where $Y_{i,t}$ and ${\hat{Y}}_{i,t}$ represent the actual observed values and the model-predicted values for sensor *i* at time t, respectively.

Considering the intricacies of industrial field environments, sensor signals are frequently susceptible to stochastic impulse noise, which may precipitate spurious alarms. To enhance reliability, a central moving average algorithm is implemented to refine the raw residual sequence:

(14) $$S_{t}=\frac{1}{W_{\mathrm{smooth}}}\sum_{W_{\mathrm{smooth}}/2}^{j=-W_{\mathrm{smooth}}/2}E_{t+j}$$

where $S_{t}$ denotes the smoothed residual intensity and $W_{\mathrm{smooth}}$ signifies the smoothing window size, which is set to 60 in this study.

Subsequently, an adaptive early-warning threshold *Th* is formulated based on the 3σ criterion, leveraging the statistical mean *u* and standard deviation σ derived from the smoothed residuals within the offline training set:

(15) Th=μ+3σ

In the online monitoring phase, a deviation is identified whenever the calculated $S_{t}$ surpasses the predefined threshold *Th*, thereby triggering an automated anomaly alert. This mechanism effectively synergizes detection sensitivity with robust noise suppression.

## 4. Experimental Validation and Analysis

**Figure 5:**
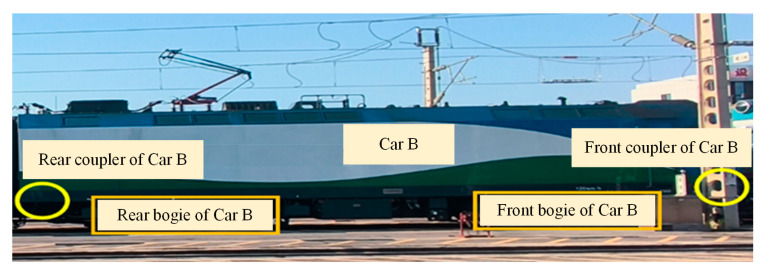
Car B of the heavy-haul train.

To evaluate the efficacy of the proposed methodology, empirical data analysis was performed by leveraging a heavy-haul train condition monitoring project. Car B of the heavy-haul train is illustrated in Figure 5.

### 4.1. Sensor Layout and Dataset Partitioning

**Figure 6:**
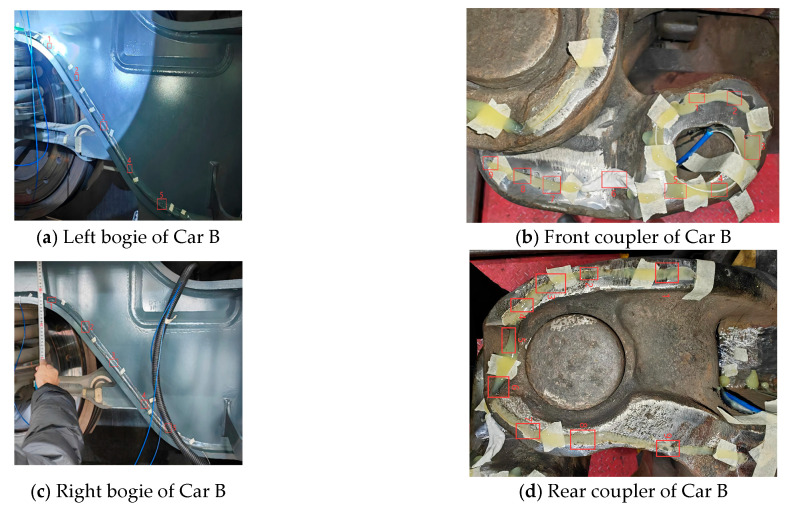
Schematic illustration of the sensor layout for the coupler and draft gear system on Car B of the heavy-haul train.

By integrating the mechanical response mechanisms of the coupler and draft gear system with signal quality requirements, this study selected 30 pivotal sensor points on Car B of the heavy-haul train to construct high-dimensional feature vectors. The physical distribution of these measurement points is illustrated in Figure 6 and detailed in Table 2. The monitoring locations encompass critical components, including strain gauges on the front and rear coupler bodies, the knuckles, pin holes, and concave platforms within the force transmission hubs, as well as the primary and secondary springs in the bogie suspension zones. Furthermore, draw-wire displacement sensors were deployed to characterize the relative longitudinal movement of the system. Collectively, these sensors constitute a spatial monitoring network governed by robust topological constraints.

The experimental dataset was derived from authentic online service records. Specifically, continuous data recorded from 3 December 2025 to 6 January 2026, during which the coupler remained in a healthy operational state, were utilized to establish the normal linkage baseline. The data used for training, normalization, and validation was derived from real-world sensor readings collected during the operational service of the heavy-haul train. Raw data underwent preprocessing to handle missing values and applied Z-score normalization to eliminate numerical disparities arising from the different physical dimensions of the sensors. The dataset was then partitioned, with 90% used for training and the remaining 10% used for testing. This approach facilitates a rigorous evaluation of the model’s predictive accuracy and generalization performance on normal operational data.

In addition to the training and test sets, a small portion, 10%, of the training data was further reserved as a validation set. This validation set was used during the training process to monitor the model’s performance and ensure it was not overfitting to the training data. The loss curve on the validation set was tracked alongside the training loss to evaluate the model’s generalization ability. It is important to note that the introduction of this validation set does not affect the original 90% training and 10% test set split; it was simply used as an additional tool for model tuning and performance evaluation.

Due to the complexity of the field working environment, the raw data exhibit localized missing values and timestamp discontinuities. To ensure the quality and physical continuity of the input data, linear interpolation was employed to fill sporadic null values, and Z-score normalization was applied to eliminate numerical disparities arising from different physical dimensions. Addressing the discontinuities caused by train stops or communication interruptions, a time interval threshold of 300 s was established to segment the original time series into multiple continuous data fragments. Subsequently, within each continuous segment, a sliding window containing 60 time steps was extracted at a fixed step size as the input feature, with the data at the immediate subsequent time step serving as the prediction target. This processing mechanism effectively prevents erroneous concatenation across discontinuous intervals, thereby constructing spatio-temporal sample sequences that conform to actual physical evolutionary laws.

In this study, the experimental data was aligned with the onboard LKJ data, ensuring accurate synchronization of sensor data with the operational timeline. This allows us to track both the train route and the corresponding load information during the experiment, which is crucial for obtaining reliable wear data from the system components. We utilized fiber Bragg grating (FBG) strain sensors for data collection. A total of 38 sensor measurement points were deployed across the single coupler. The system operated at a sampling frequency of 100 Hz. Each sensor point transmitted 4-byte wavelength data, and after quantization and calculation, the real-time output data volume was approximately 15,200 bytes (14.84 KB/s) per second. The data transmission and storage load are manageable, ensuring the system’s operational feasibility.

Regarding the selection of sensor points, we carefully chose 30 pivotal measurement locations based on their spatial coupling significance, as determined by the NMI-based sensor association matrix. The current configuration of 30 sensors strikes an optimal balance between sensor coverage and computational efficiency. While future work could investigate the impact of varying the number or distribution of sensors, the present configuration ensures high model accuracy without unnecessary complexity. The dimensionality reduction process, guided by NMI, inherently filters out weakly correlated or redundant sensors, focusing on those that provide the most meaningful contributions to the model’s performance.

**Figure 7:**
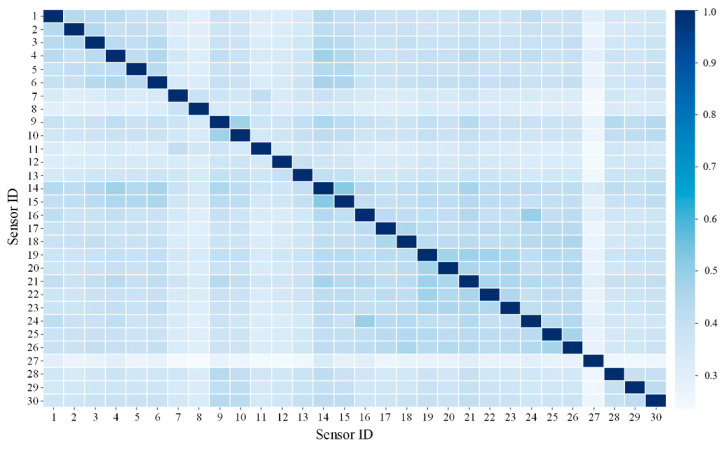
Heatmap of the spatial nonlinear coupling characteristics among sensors.

To precisely extract the spatial physical topology of the coupler and draft gear system while eliminating weakly correlated redundant noise, NMI was utilized to quantify the nonlinear coupling intensity among the 30 initial sensor points. The global sensor association matrix calculated based on the healthy training set is illustrated in Figure 7.

Figure 7 illustrates the global NMI-based association matrix constructed from all 30 sensors under healthy operating conditions. Each element in this matrix represents the normalized mutual information between a pair of sensors, where higher values indicate stronger nonlinear coupling and closer physical interaction between the corresponding measurement points. Therefore, this matrix can be interpreted as a quantitative representation of the overall spatial correlation structure and mechanical linkage characteristics within the coupler and draft gear system.

Owing to the force transmission characteristics of the internal mechanical structure of the coupler, sensors located within the same rigid transmission hub or adjacent suspension zones exhibit potent nonlinear synergistic responses, whereas the correlations between distant sensors remain relatively weak.

To quantitatively evaluate the contribution of each sensor to the overall spatial coupling structure, the cumulative association weight of each node was calculated by summing its NMI values with all other sensor nodes in the global association matrix. Sensors with higher cumulative association degrees were considered to play a more dominant role in reflecting the principal mechanical transmission paths and critical physical interactions within the coupler system.

Based on the global association matrix shown in Figure 7, this cumulative weighting process effectively identifies the most representative sensors in terms of global coupling significance. Accordingly, the dimensionality reduction from 30 sensors to 12 core sensors is directly derived from the information contained in Figure 7.

**Figure 8:**
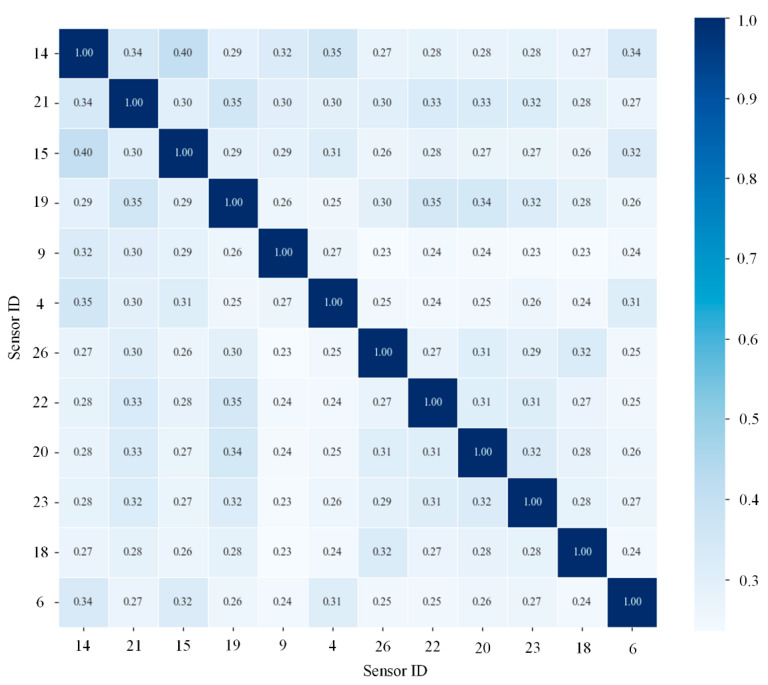
Normalized association matrix of the 12 core sensors.

To enhance the efficiency of spatial feature aggregation and the robustness against noise, the dimensionality was reduced based on the sum of global association weights for each node. Ultimately, the 12 core monitoring variables with the highest cumulative association degrees were extracted, and their refined spatial association matrix was reconstructed, as depicted in Figure 8 and detailed in Table 3.

Figure 8 presents the refined NMI association matrix constructed using the selected 12 sensors, which can be regarded as a subgraph extracted from the global matrix in Figure 7. Compared with Figure 7, this reduced matrix preserves the most critical spatial coupling relationships while eliminating weakly correlated connections, thereby forming a compact yet physically meaningful topology for subsequent graph convolution operations.

It should be emphasized that Figure 8 is not a confusion matrix for classification evaluation, but a normalized mutual information (NMI)-based association matrix describing the nonlinear coupling relationships among the selected sensors. The diagonal elements are equal to 1 because each sensor is perfectly correlated with itself after normalization, while the off-diagonal elements represent the strength of nonlinear interactions between different sensor pairs. Therefore, the matrix should be interpreted as a spatial correlation structure rather than a classification result.

This selection strategy effectively retains the most informative and physically meaningful sensors while filtering out weakly correlated and redundant variables, thereby improving computational efficiency and enhancing the robustness of the proposed model. These 12 nodes constitute the fixed graph topology for spatial convolution within the NMI-STGNN.

### 4.2. Condition Monitoring Based on Normal Service Data

**Figure 9:**
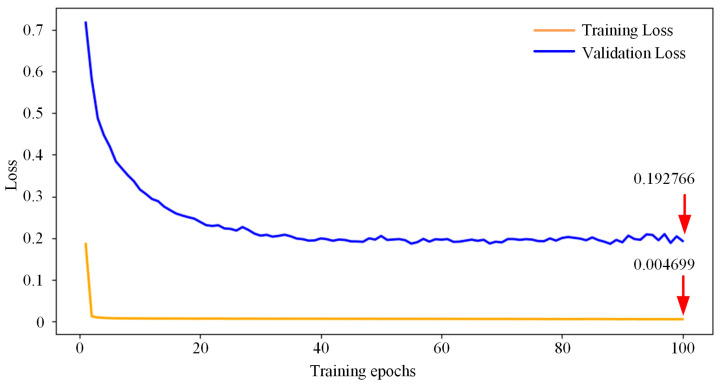
Training and validation convergence curves of the proposed model.

Once the graph structure was finalized, the healthy spatio-temporal samples were utilized for model training. The Adam optimizer was employed with a total training duration of 100 epochs. The resulting MSE convergence curve is shown in Figure 9. Both the training loss and validation loss curves are depicted to provide a more comprehensive evaluation of the model’s performance. During the initial training phase, the training loss decreased precipitously, while the validation loss also showed a consistent decrease, stabilizing asymptotically around 0.004699. This indicates that the model has sufficiently characterized the dynamic evolutionary patterns under normal working conditions. The comparison between both curves demonstrates the model’s ability to generalize well without overfitting, as the validation loss stabilizes similarly to the training loss.

**Figure 10:**
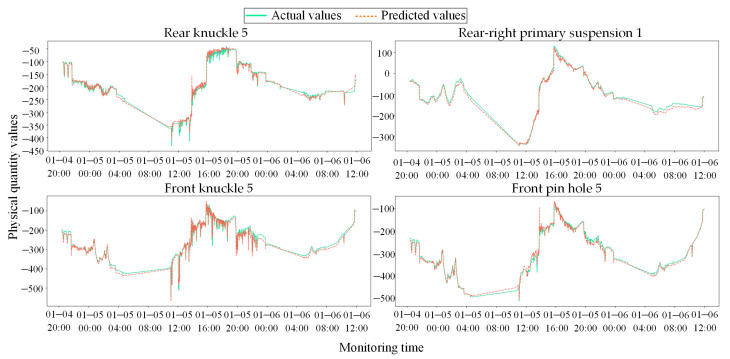
Comparison between actual physical responses and predicted results for core sensor nodes.

To evaluate the effectiveness of the model in learning the normal linkage laws of the coupler system, forward predictions were executed on the test set, followed by an inverse-standardization process. The fitting performance between the actual physical measurements and the predicted values for selected core sensor nodes is presented in Figure 10.

Under the complex and volatile working conditions of heavy-haul trains, the raw signals captured by sensor nodes exhibit high-frequency and strenuous nonlinear fluctuations. Nevertheless, the predicted signals generated by the model track the trajectories of the actual values faithfully across multiple temporal scales. High fitting precision is maintained both in stable regions with low-frequency trends and during high-frequency transients induced by alternating load impacts. These results demonstrate that the proposed STGNN effectively decouples and internalizes the robust physical topological constraints and dynamic latency characteristics inherent in the internal components of the coupler during normal service, thereby laying a solid groundwork for establishing a reliable anomaly residual baseline.

Upon confirming the high-precision state prediction capability, the model was deployed on the complete test set, which included unknown condition evolutions and potential physical degradation, to verify its anomaly early-warning performance. The model first calculates the RMSE of the test sequences. To mitigate the interference of stochastic high-frequency noise in the field on the warning mechanism, a central moving average algorithm was introduced to smooth and denoise the raw residuals. Subsequently, the early-warning threshold was adaptively established according to the 3σ criterion, utilizing the mean and standard deviation derived from the data distribution of the healthy training set.

**Figure 11:**
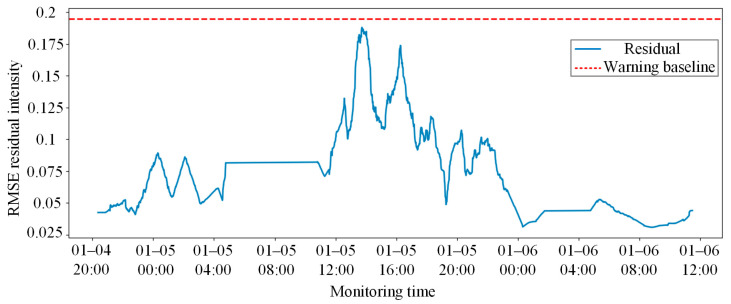
Anomaly early-warning results of the coupler and draft gear system based on smoothed residuals and the 3σ criterion.

As illustrated in Figure 11, the solid blue line represents the smoothed system composite residual intensity, while the red dashed line signifies the 3σ healthy baseline established offline. During the initial monitoring period, the system residuals fluctuate steadily below the threshold, indicating that the mechanical responses of the coupler components conform to the normal spatial linkage laws and no false alarms were triggered.

### 4.3. Comparative Experiments

To further verify the superiority of the proposed model under complex working conditions, a classic CNN-LSTM network from the field of time-series prediction was selected as the baseline model for comparative analysis.

CNN-LSTM is selected as the baseline model mainly because this architecture represents a classic paradigm for modeling spatio-temporal features in multi-dimensional sensor signals. Specifically, CNN is capable of extracting local spatial distribution patterns among different sensors, while LSTM is employed to capture the long-term temporal dependencies embedded in sequential monitoring data. By comparison with this conventional spatio-temporal modeling approach, the performance advantages of the graph structure proposed in this study can be more intuitively validated in characterizing complex mechanical topological relationships and processing data defined on non-Euclidean spaces.

Regarding parameter settings, this baseline model maintained strictly identical input specifications to the methodology proposed in this study. The specific architecture included a single 1D-CNN layer with 32 output channels and a kernel size of 3, utilizing same padding to preserve complete temporal information. This was followed by two LSTM layers with a hidden dimension of 32 and a dropout rate of 0.3 to prevent overfitting, with the final prediction results mapped via a fully connected layer. The training phase similarly employed MSE as the loss function, utilizing the Adam optimizer with a learning rate of 0.001 and a batch size of 64 over 100 epochs.

**Figure 12:**
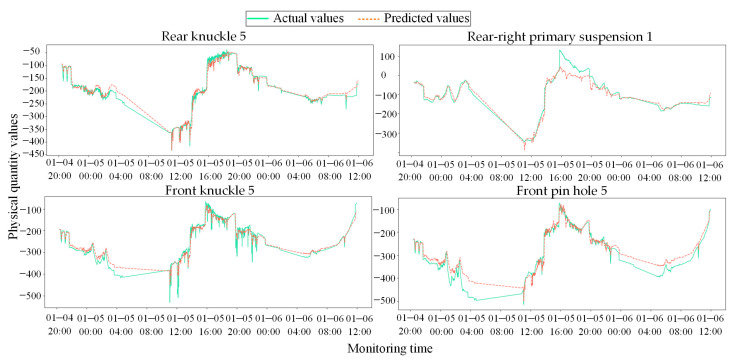
Comparison between actual physical responses and predicted results for core sensor nodes using the CNN-LSTM model.

The comparison between the actual responses of core sensor nodes and the predicted results of the CNN-LSTM model is illustrated in Figure 12. It can be observed that while CNN-LSTM possesses a degree of temporal modeling capability and can track the overall low-frequency trends of the signals, it exhibits significant fitting bias and lag when encountering high-frequency nonlinear transients induced by heavy-haul alternating impacts. This deficiency stems from its lack of effective perception regarding the spatial physical topology and synergistic constraints among the sensors.

**Figure 13:**
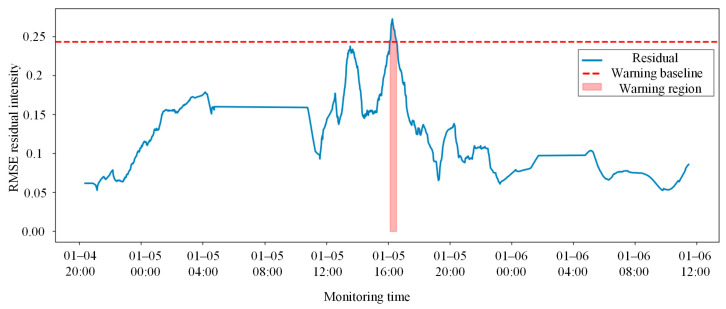
Anomaly early-warning results of the coupler and draft gear system based on smoothed residuals and the 3σ criterion using the CNN-LSTM model.

The aforementioned inaccuracies in prediction directly weaken the reliability of the subsequent healthy baseline construction. The anomaly early-warning results for the CNN-LSTM model, presented in Figure 13, indicate that the intense fluctuations in prediction residuals under normal conditions lead to an elevated alarm threshold calculated via the 3σ criterion, which significantly reduces the noise robustness of the system. During the actual monitoring phase, the residual curve of this model is highly unstable within healthy intervals and is susceptible to triggering false alarms due to local high-frequency signal interference. For instance, a prominent spurious alarm was generated by this model during a normal service period at 16:00 on 5 January 2026.

These comparative results demonstrate that relying solely on pure sequence models makes it difficult to fully decouple the complex physical mechanisms of the heavy-haul train coupler and draft gear system. The introduction of NMI and STGNN to jointly mine the spatial topology and temporal evolutionary characteristics of multi-dimensional sensor signals is significantly necessary for enhancing the state-tracking accuracy and warning robustness of the system.

To quantitatively evaluate the predictive performance of the models, this study selected MAE, RMSE, and R^2^ as the primary evaluation metrics. Among these, MAE is utilized to reflect the physical magnitude of the deviation between predicted values and actual observations. RMSE exhibits higher sensitivity to significant outliers within the prediction sequence and is employed to assess the stability of the model. R^2^ measures the goodness of fit regarding data fluctuations, where a value closer to 1 indicates a more profound capacity of the model to capture complex nonlinear features:

(16) $$MAE=\frac{1}{n}\sum_{i=1}^{n}\left|y_{i}-\hat{y_{i}}\right|$$

(17) $$RMSE=\sqrt{\frac{1}{n}\sum_{i=1}^{n}{\left(y_{i}-\hat{y_{i}}\right)}^{2}}$$

(18) $$R^{2}=1-\frac{\sum {\left(y_{i}-\hat{y_{i}}\right)}^{2}}{\sum {\left(y_{i}-\bar{y}\right)}^{2}}$$

It should be noted that both MAE and RMSE retain the same physical units as the original sensor measurements after inverse normalization. In this study, since the monitored signals are collected from FBG strain sensors, the units of MAE and RMSE are expressed in macrostrain (με), thereby directly reflecting the magnitude of prediction errors in terms of actual mechanical deformation.

Detailed performance data are presented in Table 4.

The NMI-STGNN proposed in this study demonstrates significantly superior predictive accuracy compared to the baseline CNN-LSTM across the four core sensor nodes. As detailed in Table 4, both MAE and RMSE for NMI-STGNN exhibit substantial decreases across all measurement points. Notably, at the Front pin hole 5 measurement point, which is significantly influenced by complex working conditions, the proposed method reduces the MAE from 22.7705 to 7.7411, representing a remarkable error reduction rate of 66.0%. Furthermore, the R^2^ metrics for all core nodes remain stable above 0.96, reaching a maximum of 0.9875, which demonstrates an exceptional capacity for nonlinear feature fitting and dynamic tracking. This significant enhancement validates the superiority of the proposed approach in extracting spatial interactions and temporal dynamic evolutionary characteristics of equipment states. By reconstructing the spatial physical topology of the coupler system via NMI, the model establishes a more precise and noise-robust health monitoring baseline compared to pure sequence models when processing alternating load impacts from heavy-haul trains.

### 4.4. Monitoring Based on Abnormal Operational Data

To verify the recognition capability of the proposed methodology in real mechanical degradation scenarios, this section conducts experiments using empirical data collected from 3 December to 18 December 2025. Building upon the original 30 sensors, the Rear knuckle 9 measurement point was added to construct a 31-dimensional high-dimensional feature vector. Field records indicate that due to abnormal alternating impact loads and localized stress concentration, this measurement point experienced a significant strain surge around 05:00 on 18 December 2025.

**Figure 14:**
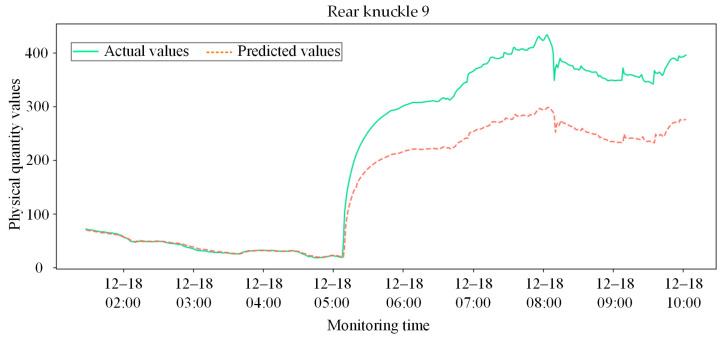
Comparison between actual physical responses and predicted results for core sensor nodes during the abnormal evolution phase.

The experimental data were strictly partitioned chronologically, with the initial 95% of healthy samples used to establish the system baseline and the remaining 5% covering the abnormal evolution period reserved as the test set. The prediction comparison results for core measurement points are presented in Figure 14.

During the initial period of the test set where no anomalies occurred, the actual signals of each node maintained a high degree of fit with the predicted signals, indicating that the model precisely internalized the normal spatio-temporal evolutionary laws of the coupler system. However, near 05:00 on 18 December the abnormal deformation of the local physical structure disrupted the original spatial topological constraints within the coupler. Consequently, the actual monitoring values of affected points, such as Rear knuckle 9, exhibited strenuous nonlinear deviations. At this juncture, the model continued to perform inference based on spatial linkage weights derived from the healthy state, leading to a pronounced residual separation between the predicted and actual values. This feature separation serves as the core criterion for identifying early equipment faults.

**Figure 15:**
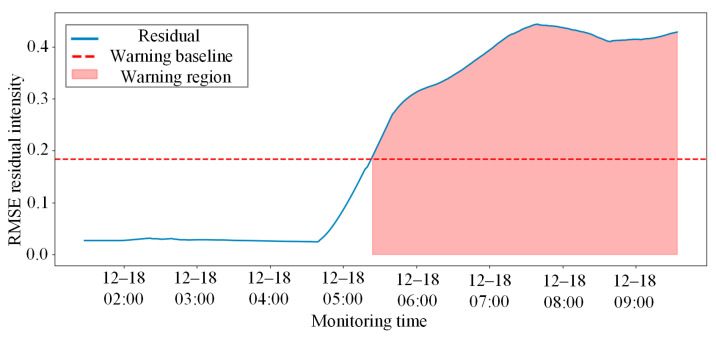
System anomaly early-warning results based on smoothed residuals and the 3σ criterion under real working conditions.

To quantitatively evaluate the health status of the entire coupler system and filter out local high-frequency noise interference, the model performs a moving average smoothing of the RMSE residuals of the test set. The adaptive early-warning threshold is established using the 3σ criterion, based on the residual distribution of the training set. This threshold represents the upper bound of the normal operational range, with any residual exceeding this value indicating an anomaly. The final system-level anomaly monitoring results, as shown in Figure 15, are based on this threshold.

During the early phase of the test set, the system composite residual intensity fluctuated steadily below the 3σ warning baseline, and no false alarms were triggered. As time progressed to approximately 05:00 on 18 December, the residual curve exhibited two sharp mutation peaks that substantially surpassed the warning baseline, triggering an automated system alert. This alarm interval aligns perfectly with the actual physical timestamp when the rear knuckle experienced a severe abnormal response in the field. These comparative experiments sufficiently demonstrate that the proposed methodology can not only sensitively capture global spatial linkage failures caused by single-point physical degradation but also effectively resist noise interference under complex conditions, achieving intelligent state early-warning with high sensitivity and low false alarm rates for heavy-haul train coupler systems.

**Figure 16:**
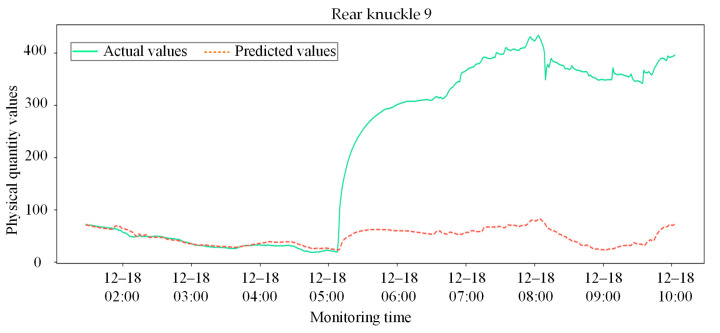
Comparison between actual physical responses and predicted results for core sensor nodes during the abnormal evolution phase using CNN-LSTM.

**Figure 17:**
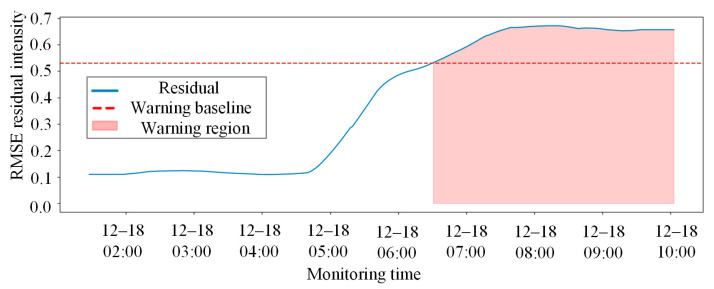
System anomaly early-warning results based on smoothed residuals and the 3σ criterion under real working conditions using CNN-LSTM.

Figure 16 and Figure 17 present the system-level anomaly early-warning results of the CNN-LSTM baseline model under real working conditions. As shown in Figure 16, the actual physical response begins to exhibit severe abnormal deviation precisely at 05:00 on 18 December. However, the predicted values generated by the CNN-LSTM completely fail to capture this mutation, remaining stubbornly flat. Consequently, as depicted in Figure 17, the system’s residual intensity does not cross the warning baseline to trigger an alarm until approximately 06:30. This significant delay of an hour and a half poses a severe safety risk in practical heavy-haul train operations. The poor performance of the CNN-LSTM model can be attributed to two main factors. First, without the NMI-based feature selection to isolate core sensors, the model processes all variables indiscriminately, leading to severe information redundancy and noise interference that dilute critical anomaly signals. Second, as a pure sequence model, CNN-LSTM inherently lacks the capacity to extract spatial synergistic constraints among multi-dimensional sensors, rendering it extremely insensitive to early, sudden fault mutations.

**Figure 18:**
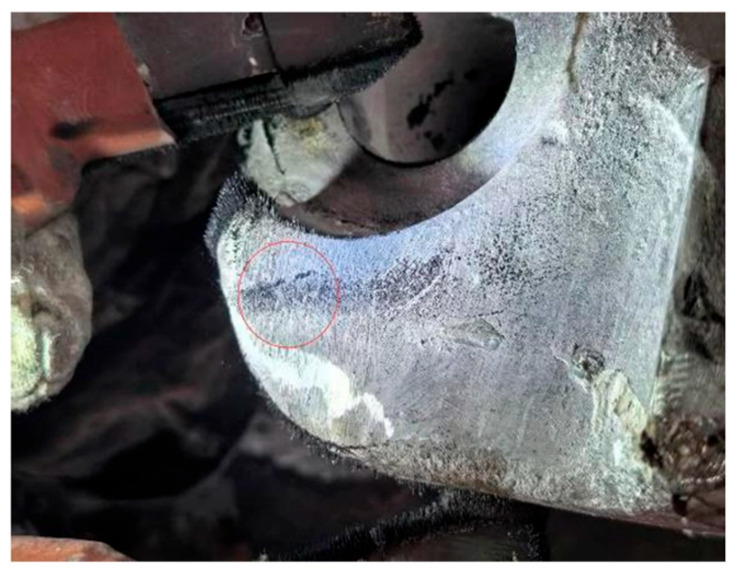
Physical photograph of the magnetic particle inspection of the knuckle on 23 December 2025.

On 23 December 2025, field inspectors performed a magnetic particle inspection on the knuckle based on the system warning information, and Figure 18 displays the physical images from the inspection process. The inspection conclusions confirmed that the initiation site of the fatigue crack discovered during the process was highly consistent with the location of the 9th sensor point, where the strain data had previously exhibited severe deviation in the monitoring system. This precise spatial correspondence indicates that the substantial damage to the internal physical structure of the knuckle disrupted the local stress balance, serving as the direct cause of the nonlinear mutations in the sensor features. This field validation via magnetic particle inspection not only substantiates the acute capability of the proposed model to capture early weak crack signals but also provides a reliable physical criterion and closed-loop data support for the intelligent operation and maintenance of coupler systems.

## 5. Conclusions

To address the safety monitoring requirements of heavy-haul train coupler and draft gear systems under complex alternating loads, this study proposes an anomaly monitoring methodology integrating NMI and STGNN. The main conclusions are summarized as follows:

(1) NMI was introduced to effectively quantify the nonlinear physical coupling intensity among multi-source sensors. By sparsifying the fully connected matrix, interference from weakly correlated noise was successfully eliminated, and a spatial physical topology reflecting the internal mechanical linkage laws of the coupler was reconstructed, providing accurate structural support for spatial feature extraction.
(2) A deep learning framework fusing GCN, GRU and 1D-CNN with residual connections is constructed. The model enables collaborative mining of spatial correlations and temporal evolution patterns in monitoring signals, thereby effectively capturing the complex physical topology constraints and dynamic time-lag characteristics inherent in the coupler and draft gear system during regular operation.
(3) An early-warning mechanism based on predictive residual smoothing and the adaptive 3σ criterion was established. By applying moving average denoising to the residual sequence, the anti-interference capability of the system in complex industrial environments was significantly enhanced, achieving an effective balance between warning sensitivity and noise robustness.
(4) Field data validation indicates that the NMI-STGNN model achieves significantly higher prediction accuracy than the traditional CNN-LSTM model. For all key sensor nodes, the MAE and RMSE are notably reduced, while the coefficient of determination R^2^ stably maintains above 0.96, which effectively suppresses false alarms under normal operating conditions.
(5) In real fault cases, the anomalous nodes identified by the model highly coincide with the fatigue crack initiation sites discovered via magnetic particle inspection, confirming the acute capability of this method to capture early faint anomaly signals. This research provides a reliable, physically consistent, data-driven solution for the real-time monitoring and condition-based maintenance of critical heavy-haul train components.

## Figures and Tables

**Table 1:** The parameters of the NMI-STGNN in this article.

Network	Layer	Filter Number/Kernels Size/Padding	Output Size
Input layer	Raw temporal signal	1/60/12	BS × 60 × 12
Graph Construction	NMI, Top-K	12 × 12/K = 5	12 × 12
Spatio-Temporal Block 1	GCN, GRU, ReLU, Residual	GCN:32-1; GRU:384-384	BS × 60 × 12 × 32
Spatio-Temporal Block 2	GCN, GRU, ReLU, Residual	GCN:32-32; GRU:384-384	BS × 60 × 12 × 32
Fusion layer	Concat, 1 × 1 Conv, ReLU	33-64-1	BS × 12
Training config	MSE Loss, Adam Optimizer	LR: $1\times 10^{-4}$; BS:64; Epochs:100	BS × 12
Anomaly Detection	3σ Threshold, Sliding Smoothing	Win:60	BS × 1

BS: Batch Size; LR: Learning Rate; Conv: Convolution; ReLU: Rectified Linear Unit.

**Table 2:** Description of the 30 sensor measurement points utilized in the experiment.

Sensor ID	Measurement Point Name	Sensor ID	Measurement Point Name
1	Rear coupler body 1	16	Front concave platform 1
2	Rear coupler body 5	17	Front concave platform 5
3	Rear coupler body 9	18	Front concave platform 9
4	Rear pin hole 1	19	Front pin hole 5
5	Rear pin hole 5	20	Front pin hole 9
6	Rear pin hole 9	21	Front knuckle 1
7	Rear-left primary suspension 1	22	Front knuckle 5
8	Rear-left primary suspension 5	23	Front knuckle 9
9	Rear-right primary suspension 1	24	Front coupler shank 1
10	Rear-right primary suspension 5	25	Front coupler shank 5
11	Rear-left secondary suspension 1	26	Front coupler shank 9
12	Rear-left secondary suspension 5	27	Draw-wire displacement 2
13	Rear-left secondary suspension 9	28	Rear-right secondary suspension 1
14	Rear knuckle 1	29	Rear-right secondary suspension 5
15	Rear knuckle 5	30	Rear-right secondary suspension 9

**Table 3:** The 12 most representative sensors selected via NMI.

Sensor ID	Measurement Point Name	Sensor ID	Measurement Point Name
14	Rear knuckle 1	26	Front coupler shank 9
21	Front knuckle 1	22	Front knuckle 5
15	Rear knuckle 5	20	Front pin hole 9
19	Front pin hole 5	23	Front knuckle 9
9	Rear-right primary suspension 1	18	Front concave platform 9
4	Rear pin hole 1	6	Rear pin hole 9

**Table 4:** Quantitative comparison of predictive performance between the proposed methodology and CNN-LSTM for core sensors.

Measurement Point Name	Model Type	MAE (με)	RMSE (με)	R^2^ (-)
Rear knuckle 5	NMI-STGNN	8.8274	14.1751	0.9699
	CNN-LSTM	13.9001	13.9001	0.9379
Front knuckle 5	NMI-STGNN	6.6304	9.7280	0.9838
	CNN-LSTM	10.0819	14.3601	0.9647
Rear-right primary suspension 1	NMI-STGNN	8.6493	10.8754	0.9873
	CNN-LSTM	16.1557	25.3166	0.9313
Front pin hole 5	NMI-STGNN	7.7411	10.4974	0.9875
	CNN-LSTM	22.7705	29.3084	0.9023

## Data Availability

The dataset is available on request from the authors.
